# Characterization of volatile fatty-acid utilization in *Escherichia coli* aiming for robust valorisation of food residues

**DOI:** 10.1186/s13568-020-01121-4

**Published:** 2020-10-17

**Authors:** Gustav Sjöberg, Martin Gustavsson, Antonius J. A. van Maris

**Affiliations:** Department of Industrial Biotechnology, School of Engineering Sciences in Chemistry, Biotechnology and Health, KTH Royal Institute of Technology, Stockholm, Sweden

**Keywords:** Volatile fatty acids, VFA, Food waste, *Escherichia coli*, Anaerobic digest

## Abstract

Valorisation of food residues would greatly benefit from development of robust processes that create added value compared to current feed- and biogas applications. Recent advances in membrane-bioreactor-based open mixed microbial cultures, enable robust conversion of fluctuating streams of food residues to a mixture of volatile fatty acids (VFAs). In this study, such a mixed stream of VFAs was investigated as a substrate for *Escherichia coli*, a well-studied organism suitable for application in further conversion of the acids into compounds of higher value, and/or that are easier to separate from the aqueous medium. *E. coli* was cultured in batch on a VFA-rich anaerobic digest of food residues, tolerating up to 40 mM of total VFAs without any reduction in growth rate. In carbon-limited chemostats of *E. coli* W3110 ΔFadR on a simulated VFA mixture, the straight-chain VFAs (C_2_-C_6_) in the mixture were readily consumed simultaneously. At a dilution rate of 0.1 h^−1^, mainly acetic-, propionic- and caproic acid were consumed, while consumption of all the provided acids were observed at 0.05 h^−1^. Interestingly, also the branched isovaleric acid was consumed through a hitherto unknown mechanism. In total, up to 80% of the carbon from the supplied VFAs was consumed by the cells, and approximately 2.7% was excreted as nucleotide precursors in the medium. These results suggest that VFAs derived from food residues are a promising substrate for *E. coli*.

## Key points

- Volatile fatty acids from food waste are a suitable carbon source for *E. coli*.
- Consumption of all supplied volatile fatty acids was observed in chemostats.

## Introduction

Approximately one third of the food produced for humans is lost as waste before consumption (FAO 2011). Together with actions to decrease these losses, valorisation of the inevitable remaining residues is preferred over dumping in landfills, where anaerobic bacteria decompose the waste into the potent greenhouse-gas methane. Similar natural consortia of bacteria can also convert a wide-range of different, fluctuating food residues to methane under more controlled conditions in a process called anaerobic digestion (Keller and Surette 2006; Brenner et al. 2008; Smid and Lacroix 2013). The resulting biogas is a drop-in replacement for natural gas, providing energy for propulsion, electricity or heat (Scarlat et al. 2018). However, the value of biogas is relatively low and is often subsidised to benefit from the reduction in waste volumes and greenhouse gas emissions in addition to the production of green energy and fuels (Bartolini et al. 2017).

Like the open mixed cultures of anaerobic digestion, alternative processes for valorising food residues must be robust and able to cope with the fluctuations of the incoming residues, while yielding stable value-added product streams. This eliminates the use of pure cultures, since they cannot deal with the complex and fluctuating compositions of mixed food waste and are additionally economically undesirable and difficult to sterilize. Although production of methane is thermodynamically favoured, by operating anaerobic digestion under conditions that are unfavourable to methanogens, such as reduced pH, reduced hydraulic retention time, or increased temperature (Lee et al. 2014), formation of volatile fatty acids (VFAs) is promoted instead.

Volatile fatty acids (VFAs) are carboxylic acids with between two and six carbon atoms, produced as intermediates during digestion of organic material and have higher-value applications than the otherwise produced biogas. These applications include polymers (e.g. vinyl- and cellulose esters), pharmaceuticals, fragrances and flavours (i.e. various esters), food and feed supplements, crop protection and disinfecting products (Kubitschke et al. 2014). Recent advances in application of membrane bioreactors for anaerobic digestion have simplified the recovery of VFAs from the broth, by allowing continuous removal of a clear liquid fraction from the solids (Trad et al. 2015; Wainaina et al. 2019). However, the total VFA concentration in the effluent remains low, and thus distillation or extraction of the individual acids cannot compete with current, fossil-based production methods (Kleerebezem et al. 2015). As an alternative to purifying VFAs for direct use, they can be further valorised to a set of valuable products by microbial conversion (Kleerebezem et al. 2015). Compounds that can be readily produced include, but are not limited to: polymers, alcohols, hydroxyacids, lipids and esters (Chang et al. 2010; Lee et al. 2014). Such conversions allow VFAs to become a platform intermediate that eliminates the variation of food residues, while preserving most of the contained electrons of this complex substrate. Unlike the original food residues, the resulting VFA mix is composed of a fixed set of compounds with potential as a carbon- and energy source for pure cultures of microorganism able to produce value-added chemicals, as well as direct microbial conversion into value added compounds.

*Escherichia coli* is a well-established bacterial host and has been studied extensively for proof-of-concept production of numerous compounds (Calero and Nikel 2019). In addition to the wide variety of possible (engineered) products, *E. coli* consumes a wide range of substrates, including all the straight chain fatty acids from two to six carbons (Salanitro and Wegener 1971). However, anaerobic digestion produces a mixture of these VFAs and the simultaneous consumption of these has not been previously characterized in *E. coli*. Additionally, the limits of tolerance of *E. coli* to this mixture of weak organic acids, which can diffuse through the membrane and uncouple the membrane potential (Baronofsky et al. 1984; Gabba et al. 2020), is unknown.

The aim of this study was to investigate the potential of *Escherichia coli* as a platform microorganism for valorisation of the relatively clean and defined VFA streams originating from membrane-bioreactor-based anaerobic digestion (Wainaina et al. 2019). Determination of the requirements for co-consumption of VFAs by *E. coli* is a first step towards engineering the subsequent metabolism for their valorisation. First, the limits of tolerance, (co)consumption and growth of *E. coli* on a VFA solution from an anaerobic digest of food residues was characterized in batch shake flask cultures. Subsequently, co-consumption of the VFAs was investigated in carbon-limited chemostat cultures.

## Methods

### Strains

To assess VFA consumption and tolerance of *E. coli*, the reference strain W3110 (ATCC® 27,325™, F^−^ lambda^−^ IN(*rrnD*-*rrnE*)1 *rph*-1) and the W3110-derived W3110 ΔFadR were used. W3110 ΔFadR contains an in-frame deletion of the transcriptional regulator FadR, and was kindly provided by Prof. Sang Yup Lee. Both strains were stored at − 80 °C in minimal salts medium with 5 g L^−1^ glucose and 25% glycerol.

### Minimal salt media

All experiments were carried out in minimal salts medium consisting of 5 g L^−1^ (NH_4_)_2_SO_4_ (Merck, Darmstadt, Germany), 1.6 g L^−1^ KH_2_PO_4_ (VWR International, Leuven, Belgium), 0.5 g L^−1^ diammonium citrate (Merck) and 6.6 g L^−1^ Na_2_HPO_4_·2H_2_O (VWR International), which was sterilized by autoclaving for 20 min at 121 °C. 1 M MgSO_4_ and trace element stock solution (Sandén et al. 2003) were autoclaved separately (20 min at 121 °C) and added to a final amount of 1 mL per liter minimal salts medium each. Carbon sources were added as described in the following sections.

### Shake flask cultivations with an anaerobic digest as carbon source

Anaerobic digest was provided by Prof. Taherzadeh (Borås University, Sweden) in two plastic bottles containing 3 L of clarified food residue digest from a previous experiment (Wainaina et al. 2019). Upon arrival, the solution was thawed, and the liquid was decanted from a rust-brown flaky precipitate, which had also been observed by Wainaina et al. (2019) after storage of the solution at -20 °C (personal communication). The clarified solution was aliquoted in smaller glass bottles and refrozen until use. Upon rethawing, the aliquots were adjusted to pH 7.0 with 1 M NaOH and then filter-sterilized through bottle-top 0.22 µm polyethersulfone filters (Corning, New York, USA). The sterile digest was diluted in autoclaved deionized water and a 10X concentrate of the minimal medium salts. For the control experiment without minimal medium, 10% anaerobic digest and 90% 88 mM MOPS (AppliChem, Darmstadt, Germany) were mixed (v/v).

Cells were prepared by thawing glycerol stocks of the desired strains and inoculating to minimal salts medium, supplemented with 5 g L^−1^ glucose (Thermo Fisher Scientific, Waltham, USA) from an autoclaved stock solution. After overnight incubation at 37 °C with 180 rpm shaking (Minitron HT Infors, Bottmingen-Basel, Switzerland), the cells had reached an optical density at 600 nm (OD_600_) between 1.0 and 2.0. Exponentially growing cells were harvested by centrifugation at 3000 g for 10 min (Avanti J-20 XP, Beckman Coulter, Brea, USA) and resuspended in the various dilutions of anaerobic digest. Baffled shake flasks were filled to 10% of their maximum volume to ensure sufficient oxygen transfer, and incubated at 37 °C with 180 rpm shaking. Samples were withdrawn regularly for determination of OD_600_ and analysis of the medium by high-performance liquid chromatography (HPLC).

### Chemostat cultivations on defined medium with VFAs as carbon source

A defined medium with the same distribution of VFAs as measured in the anaerobic digest was designed for chemostat experiments, thereby circumventing volumetric limitations of the available digest from Wainaina et al. (2019). Each carboxylic acid was added directly to 80 L of previously autoclaved minimal salts medium, to final concentrations of 860 mg L^−1^ acetic acid, 170 mg L^−1^ propionic acid, 640 mg L^−1^ butyric acid, 580 mg L^−1^ isovaleric acid, 100 mg L^−1^ valeric acid and 2500 mg L^−1^ caproic acid (≥ 99%, Sigma-Aldrich, St. Louis, USA). The minimal medium was set to pH 7.0 by addition of 1.59 g L^−1^ of NaOH (Merck).

Chemostats were run in a parallel system with 6 steam-sterilized stainless-steel stirred-tank bioreactors (GRETA, Belach Bioteknik, Skogås, Sweden). The connections to the medium tank were made with autoclaved silicon tubing through the integrated peristaltic pump heads. Each inlet pump was calibrated daily to the desired flow-rate using an inline burette, that could be filled by aspiration through a sterile air filter (Filtropur S, Sarstedt, Nümbrecht, Germany). The volume of each bioreactor was automatically maintained at 800 mL by activation of the outlet pumps by conductivity-based level sensors. Each outlet tube was attached to a sterile 20L plastic bottle for aseptic collection of broth (Nalgene, Thermo Fisher Scientific). The pH was maintained at 7.0 by automated titration with 4 M H_2_SO_4_. Starting at 500 rpm and 200 mL min^−1^ in the batch phase, the stirring speed and air flow were increased as required to maintain a dissolved oxygen tension above 30% during the chemostat phase. The cultures were monitored by aseptically withdrawing 20 mL samples with a syringe for determination of cell dry weight (CDW), OD_600_ and supernatant composition. The outlet gas from each reactor was connected through a multiplexer (Belach Bioteknik) to a 1313 Fermentation Monitor (LumaSense Technologies, Santa Clara, USA) for determination of CO_2_ concentrations.

Inoculum for all 6 bioreactors was prepared by transfer of a W3110 ΔFadR freezer stock to a sterile stainless-steel stirred-tank bioreactor (Belach Bioteknik) containing 5.0 L of minimal medium supplemented with 5 g L^−1^ of glucose. The stirrer speed was set to 1000 rpm and 5.0 L min^−1^ headspace airflow was applied. After overnight culture, the broth of the exponentially-growing culture (with an OD_600_ between 1.0 and 2.0) was centrifuged aseptically at 3000*g* for 10 min (Sorvall BIOS 16, Thermo Fisher Scientific), then resuspended in the chemostat medium with VFA before inoculation by syringe to each of the 6 bioreactors.

### Analyses

The concentrations of phosphate and ammonia in the anaerobic digest were determined spectrophotometrically using a Cedex Bio Analyzer (Roche Diagnostics, Mannheim, Germany) according to the manufacturer’s instructions.

OD_600_ was measured in a spectrophotometer (Genesys 20, Thermo Fisher Scientific) at 600 nm after dilutions to OD_600_ between 0.1 and 0.2 with a 9 g L^−1^ NaCl solution.

CDW was measured in triplicate for each sample point. 5 mL of medium was added to dried and pre-weighed glass tubes, weighed and then centrifuged at 2500*g* in a tabletop centrifuge (Z206 A, Hermle, Gosheim, Germany) for 10 min, washed once with 5 mL of 9 g L^−1^ NaCl, centrifuged again and dried overnight. The CDW (g L^−1^) was determined by dividing the dry weight of the cells by the volume of the original broth sample.

HPLC was used to analyze the composition of culture supernatants, using an Alliance 2695 system (Waters, Milford, MA, USA) equipped with a 2414 refractive index detector (Waters), a 2996 photodiode array detector (Waters), and a TCM column heater (Waters). VFAs were separated on an Aminex HPX-87H organic acid column (Bio-Rad, Hercules, CA, USA) with a mobile phase containing 0.2% phosphoric acid and 5% acetonitrile in MilliQ water, at a flow rate of 0.9 mL min^−1^. The column was maintained at 85 °C and the acids were quantified with the photodiode detector at set at 210 nm (Bell et al. 1991). Orotic acid, uracil, and thymine were separated on the same column but at room temperature, with a flow of 0.5 mL min^−1^, and with 0.008 N sulfuric acid as mobile phase. The UV spectrum, refractive index and retention times of the reference compounds were used for identification, and peaks at 210 nm were used for quantification. Tricarboxylic-acid-cycle intermediates and common *E. coli* fermentation products were quantified based on previously determined retention times and absorbance spectra. Preparative HPLC was performed under the same conditions, but with the maximum possible injection volume, 200 µL. Fractions were collected manually as they exited the photodiode detector. Each fraction of interest was dried under vacuum and dissolved in 1 mL D_2_O (Merck), before analysis by nuclear magnetic resonance (NMR). Spectra were obtained at 400 MHz with 128 scans on a Bruker Avance (Bruker Corporation, Billerica, USA). Amino acids were assayed using the AccQ-Tag method (Waters) with proprietary reagents, according to the manufacturer’s instructions. Nucleotides and nucleosides were analyzed according to the protocol developed by Childs et al. (1996).

## Results

To investigate the requirement for additional nutrients for cultivation of *E. coli*, the concentrations of VFAs, free phosphate, and free ammonia in the sample of clarified anaerobic digest of food residues were measured (Table 1). This identified that the low amounts of free phosphate and ammonia would be growth limiting compared to the VFA content. Thus, the digest was supplemented with minimal salts medium to ensure that the VFA content would be the limiting factor.

**Table 1:** Measured concentrations of key components of the anaerobic digest used in shake flask batch cultures of *E. coli*

Component	Concentration (g L^−1^)
Acetic acid	0.81
Propionic acid	0.13
Butyric acid	0.53
Isovaleric acid	0.49
Valeric acid	0.09
Caproic acid	2.01
Free phosphate	0.018
Free ammonia	0.123

### Tolerance to and consumption of VFAs from anaerobic digest by *E. coli*

**Fig. 1:**
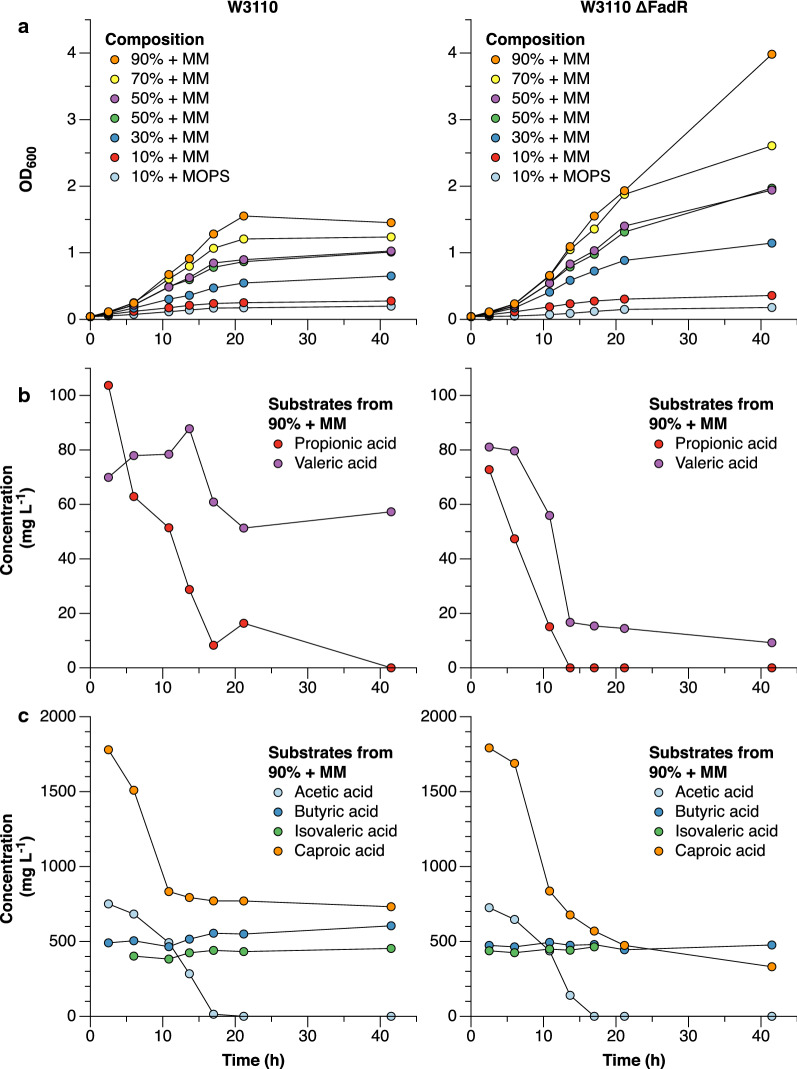
Shake flask cultivation of *E. coli* W3110 (left) and W3110 ΔFadR (right) on a VFA-rich anaerobic digest of food residues supplemented with minimal medium salts (MM) at pH 7.0, 37 °C. To assess the tolerance of the strains, the anaerobic digest was supplied in concentration increments between 10–90% (v/v) diluted with water, with constant MM concentration. A replicate experiment was performed at 50% (v/v) digest concentration.** a** Growth curves for the two strains at various dilutions, supplemented with minimal salts medium (+ MM) or MOPS buffer (+ MOPS).** b**,** c** Residual acid concentrations in the medium supplemented with 90% anaerobic digest. The concentrations from each separate culture are available in Additional file 1: Fig. S2

To assess its tolerance to the anaerobic digest, the VFA constituents of which are known growth inhibitors, *E. coli* W3110 was grown in shake flasks with dilutions of the digest (Fig. 1a). The strain grew on the anaerobic digest across the range of concentrations tested (Fig. 1a), indicating that *E. coli* is sufficiently robust towards the VFA concentrations in the current generation of membrane bioreactor anaerobic digest (Wainaina et al. 2019). As expected from the composition of the digest, W3110 did not show significant growth in a control experiment lacking minimal salts medium supplementation (10% + MOPS in Fig. 1a). After approximately 10 h, growth started to decrease and significant amounts of VFAs remained unconsumed by W3110 (Fig. 1b, c).

To de-repress pathways involved in fatty acid degradation and possibly improve VFA consumption, the fatty acid degradation transcriptional regulator (encoded by *fadR*) was deleted, and the resultant strain W3110 ΔFadR was compared to the W3110 wild type (Fig. 1). Over the first 10 h, W3110 ΔFadR showed similar tolerance to the anaerobic digest (Fig. 1a) and achieved similar initial growth rates (Additional file 1: Fig. S2). Interestingly, W3110 ΔFadR maintained growth for a longer time and reached a higher final OD_600_. This can in part be explained by a higher uptake of valeric acid and caproic acid (Fig. 1b, c), due to the lack of repression of fatty acid catabolism. However, the final OD_600_ of W3110 ΔFadR increased more than can solely be explained by the increased consumption of valeric- and caproic acid (Fig. 1a), which might be caused by consumption of a hitherto unidentified compound during the last 20 h of cultivation. Although W3110 ΔFadR consumed a larger fraction of the VFAs than W3110, consumption of valeric- and caproic acid was not complete and W3110 did not consume butyric- and isovaleric acid.

### Chemostat culture of *E. coli* on VFAs

Carbon-limited chemostat cultures are known to promote co-consumption of various carbon sources that in batch would either be consumed sequentially or not at all (Egli et al. 1993). To improve consumption of the VFAs present in the anaerobic digest (Table 1) and to characterize the co-consumption of VFAs in *E. coli* more thoroughly, the behaviour of W3110 ΔFadR was investigated in carbon (VFA)-limited chemostat cultures at dilution rates of (approximately) 0.05 and 0.10 h^−1^. Since the amounts available of the original material were insufficient, the chemostat culture was performed on a defined medium with a VFA distribution identical to the anaerobic digest (Table 1). During the start-up batch phase of the culture, it was observed that the growth rate in the defined medium was 0.140 ± 0.005 h^−1^, which is lower the initial growth rate of 0.2 h^−1^ observed in the anaerobic digest (Additional file 1: Fig. S1). The lower of the two dilution rates took more than the theoretical 5 volume changes to reach steady state, and the volume varied significantly due to foaming, which triggered the level sensor-activated outlet pump and caused a rather large standard deviation (15% relative standard deviation) on the dilution rate between replicates (Table 2).

**Table 2:** Summary of values from chemostat cultivations of W3110 ΔFadR on a defined minimal salts medium with a mix of VFAs as carbon source

D (h^−1^)	0.054 ± 0.008		0.094 ± 0.001	
CDW (g L^−1^)	2.06 ± 0.05		0.66 ± 0.05	
Y_X/S_ (g g^−1^)	0.53 ± 0.02		0.48 ± 0.04	
q_CO2_ (mmol g^−1^ h^−1^)	2.2 ± 0.4		3.9 ± 0.3	
q_Orotic acid_ (mmol g^−1^ h^−1^)	0.026 ± 0.003		0.039 ± 0.003	
Carbon balance	92% ± 7%		103% ± 6%	

	mmol g^−1^ h^−1^	mmol DR^a^ g^−1^ h^−1^	mmol g^−1^ h^−1^	mmol DR^a^ g^−1^ h^−1^
q_Acetic acid_	0.38 ± 0.05	3.0 ± 0.4	2.1 ± 0.2	16 ± 1
q_Propionic acid_	0.051 ± 0.003	0.72 ± 0.04	0.24 ± 0.03	3.4 ± 0.4
q_Butyric acid_	0.08 ± 0.02	1.5 ± 0.3	ND^b^	ND^b^
q_Isovaleric acid_	0.009 ± 0.002	0.25 ± 0.06	ND^b^	ND^b^
q_Valeric acid_	0.025 ± 0.003	0.66 ± 0.08	ND^b^	ND^b^
q_Caproic acid_	0.57 ± 0.08	18 ± 2	0.46 ± 0.05	15 ± 1
q_Total VFA_	4.8 ± 0.6 ^c^	24 ± 3	7.6 ± 0.6 ^c^	35 ± 4

The medium composition was designed from measurements of an anaerobic digest of food residues

Averages ± standard deviations. n = 6 (biological triplicates each sampled at two different timepoints separated by at least 1/D hours)

D denotes the chemostat dilution rate; q is the specific consumption rate of the indicated compound

(a) degree of reduction (Heijnen 1994)

(b) no significant consumption detected

(c) mmol carbon g^−1^ h^−1^

**Fig. 2:**
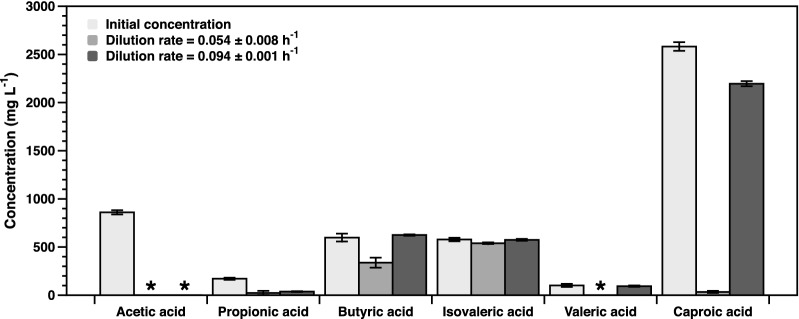
Residual substrate concentrations in aerobic chemostat cultures of W3110 ΔFadR in minimal salts medium with the indicated mix of VFAs (light grey) as carbon source at pH 7.0 and 37 °C. The bars show average concentrations with standard deviations (n = 6, biological triplicates each sampled at two different timepoints separated by at least 1/D hours) at two different dilution rates, compared to the initial concentration in the medium. * below the detection limit (10 mg L^−1^)

At a growth rate of 0.094 h^−1^, about 90% of the consumed electrons and carbon came from acetic- and caproic acid, while the remainder originated from propionic acid. At this dilution rate, only 23% ± 1% of the VFAs were consumed on a carbon basis. Whilst all the acetate was consumed, only 14% ± 1% of caproate and 73% ± 6% of propionate was consumed, whilst butyric-, isovaleric- and valeric acid were not consumed at all (Fig. 2). At 0.094 h^−1^, the consumption rate of VFAs was 7.6 ± 0.6 mmol_carbon_ g^−1^ h^−1^, which resulted in 0.66 ± 0.05 g CDW at a biomass yield on VFA of 0.48 ± 0.04 g g^−1^.

The biomass yield on consumed substrate (Y_X/S_) at a dilution rate of 0.054 h^−1^ was 0.53 ± 0.02 g g^−1^, which was not significantly different from the higher dilution rate. However, the CDW was about threefold higher at 2.06 ± 0.05 g L^−1^ for the dilution rate of 0.054 h^−1^ (Table 2). This difference is mainly explained by a higher total carbon consumption due to improved co-utilization of butyric- (44% ± 9% consumed), valeric- (97% ± 0% consumed), and caproic acid (99% ± 1% consumed) at the lower dilution rate (Fig. 2). At this lower dilution rate, the specific consumption rates of acetic- and propionic acid were reduced, due to both the lower dilution rate as well as the co-consumption of additional carbon sources (Table 2). In these conditions, most of the electrons were derived from caproic acid, while the shorter chain acids together contributed less than 25% of the degree of reduction (DR) (Heijnen 1994). Despite the increased consumption of butyric acid at 0.054 h^−1^, a substantial amount remained in the medium. In addition, at a growth rate of 0.054 h^−1^ W3110 ΔFadR consumed isovaleric acid at a significant, albeit slow, rate (Table 2). However, the contribution of this compound to the total carbon consumed was minimal (< 1% of total carbon).

Although the carbon balance only closed to 90% ± 7% at the lower dilution rate, supernatant samples tested negative for the presence of amino acids, commonly occurring fermentation products and citric acid cycle intermediates. Instead, a set of unknown peaks was detected by refractive index and UV absorbance on the carbohydrate- and organic acid column. The chromatography was repeated on a preparative scale for one of the samples and interesting fractions were collected. Based on the NMR spectra of one of the unknown fractions, it was possible to identify orotic acid as a by-product, contributing to approximately 2.7% of the carbon at both dilution rates, which improved the carbon balance to 92% ± 7%. Since orotic acid is an intermediate of nucleotide synthesis, other compounds belonging to these pathways were also sought. Thereby, trace levels of thymine and uracil were identified in the medium samples, though they did not contribute significantly to the carbon balance.

## Discussion

The aim of this study was to evaluate the use of a clarified anaerobic digest rich in VFAs as a carbon- and energy source for *E. coli*. As a first step, it was established that the toxic effects of up to 40 mM mixed VFAs, as found in the anaerobic digest, were not significant at pH 7, which is in line with previous observations on the impact of rumen VFAs (Wolin 1969) or C_6_–C_10_ carboxylic acids on *E. coli* (Royce et al. 2013). Interestingly, the observation of a higher growth rate on the anaerobic digest (0.2 h^−1^) compared to the growth on defined VFA medium in the batch phase preceding the chemostat culture (0.14 h^−1^), is also in line a previously observed growth stimulation by rumen fluid (Wolin 1969). These observations suggest that both rumen fluid and the anaerobic digest contain unidentified growth-promoting compounds.

Consumption of as many of the available VFAs as possible is another important aspect of valorisation of the VFAs from anaerobic digests. In the absence of long chain acyl-CoA (C_14_-C_18_), which is expected in the anaerobic digest, FadR represses the genes encoding the fatty acid degradation pathway (Overath et al. 1969; Cronan and Subrahmanyam 1998). By deleting FadR, the fatty acid degradation pathway, which is known to be able to beta-oxidize acids with a chain length as low as four carbon atoms (Binstock et al. 1977), was de-repressed and thereby significantly increased the fraction of VFAs that was consumed. Co-consumption of VFAs was further improved in carbon-limited chemostats, where a dilution rate of 0.054 h^−1^ allowed at least partial consumption of all the acids. Given the long time needed to achieve steady state, it is likely that adaption contributed to the additional consumption of caproic acid, and to a lesser extent also isovaleric- and butyric acid, at the lower dilution rate, whilst this was not observed at 0.094 h^−1^. The significantly higher caproic acid consumption rate at 0.054 h^−1^ compared to 0.094 h^−1^ (p = 0.01, n = 6), indicates that capacity of the uptake system was not the main limiting factor. This makes it likely that activation of the free fatty acid to the acyl-CoA was limiting the consumption of these VFAs at the higher dilution rates, as previously shown for low expression of acetyl-CoA- transferases with specificity towards butyric acid (Pauli and Overath 1972). The overall increased fraction of VFAs resulted in a higher CDW, which correlates well with previous chemostat cultures on mixed substrates (Egli et al. 1993). At the lowest dilution rate, 75% of VFAs expressed as degree of reduction came from the consumed caproic acid, whilst this was equally shared between acetic acid and caproic acid at the higher dilution rate. The Y_x/s_ expressed in g biomass formed per g VFA consumed was higher at the lower dilution rate, which seems to contradict the impact of the maintenance requirement on the biomass yield (Pirt and Hinshelwood 1965). However, when corrected for the higher degree of reduction (and thereby energy density) of caproic acid, the Y_x/s_ was 2.22 ± 0.07 g CDW mol_DR_^−1^ at 0.054 h^−1^ versus 2.7 ± 0.2 g CDW mol_DR_^−1^ at 0.094 h^−1^. This reflects the increased impact of maintenance at the lower dilution rate.

The VFA limited chemostat also yielded two unexpected results not previously observed for *E. coli*: Consumption of isovaleric acid and secretion of orotic acid. The low, but statistically significant (p = 0.0002), isovaleric acid consumption exceeds quantities that at a pH of 7 can simply accumulate intracellularly or be adsorbed by cell constituents. One or more of the acetyl-CoA-transferases expressed in the W3110 ΔFadR strain under VFA-limited conditions might show promiscuity towards isovaleric acid to produce isovaleryl-CoA. However, no dedicated isovaleryl-CoA degradation pathways are known in *E. coli*, which would require further metabolism or conversion to a currently unidentified compound through other promiscuous enzyme activities, such as the previously shown reduction of isovaleryl-CoA to 3-methylcrotonyl-CoA by AidB (Rohankhedkar et al. 2006). Although excretion of a wide range of nucleobases by *E. coli*, such as thymidine, thymine, uracil, cytosine, and guanine, has previously been observed after selection in chemostat cultures (Tsen 1994), excretion of orotic acid was not previously observed. Since orotic acid itself, and the pyrimidines derived from it, can be valuable products, it may be interesting to investigate the metabolic pathway leading to its formation and further engineer *E. coli* to improve the flux towards the product.

The ability to tolerate and grow in the anaerobic digest and to co-consume the VFAs, makes *E. coli* an interesting host for transforming VFAs to more valuable compounds and thereby valorise a diverse range of residue streams. Deletion of FadR increased the range of VFAs that were consumed in batch conditions and application of carbon-limited conditions with VFAs as the sole carbon- and energy source in chemostats showed that all substrates could (to some extent) be co-consumed. This makes it likely that the slow growth and low consumption of butyrate and isovalerate can be alleviated by laboratory evolution, for example in repeated batch- or chemostat cultivation on the substrate mix, or by engineering of the VFA consumption pathways.

## Supplementary information

**Additional file 1: Fig. S1.** Specific growth rates from the experiment presented in Fig. 1a, with the same colourcoding. **Fig. S2.** Concentrations of all quantified volatile fatty acids from the experiments presentedFig. 1a, with the same colour coding.

## Data Availability

All data generated or analysed during this study are included in this published article and its additional files.

## Abbreviations

CDW: Cell dry weight
OD_600_: Optical density at 600 nm
VFAs: Volatile fatty acids
HPLC: High performance liquid chromatography
NMR: Nuclear magnetic resonance
FadR: Fatty acid degradation transcriptional regulator (encoded by *fadR*)
Y_X/S_: The yield of cells on substrate
DR: Degree of reduction
