# Supplementary material for: Characterization of volatile fatty-acid utilization in Escherichia coli aiming for robust valorisation of food residues
Source: AMB Express. 2020 Oct 17;10:184. doi: 10.1186/s13568-020-01121-4 (PMC7568742; doi:10.1186/s13568-020-01121-4)
Supplement: Supplementary file 1 — Additional file 1: Fig. S1. Specific growth rates from the experiment presented in Fig. 1a, with the same colourcoding. Fig. S2. Concentrations of all quantified volatile fatty acids from the experiments presentedFig. 1a, with the same colour coding. [file 13568_2020_1121_MOESM1_ESM.pdf]

Applied Microbiology and Biotechnology

Supplementary material

Characterization of volatile fatty-acid utilization in *Escherichia coli* aiming for robust valorisation of food residues

Gustav Sjöberg[1], Martin Gustafsson[1], Antonius J.A. van Maris[1,]*

[1] Department of Industrial Biotechnology, School of Engineering Sciences in Chemistry, Biotechnology and Health, KTH Royal Institute of Technology, Stockholm, Sweden

* Corresponding author, tonvm@kth.se

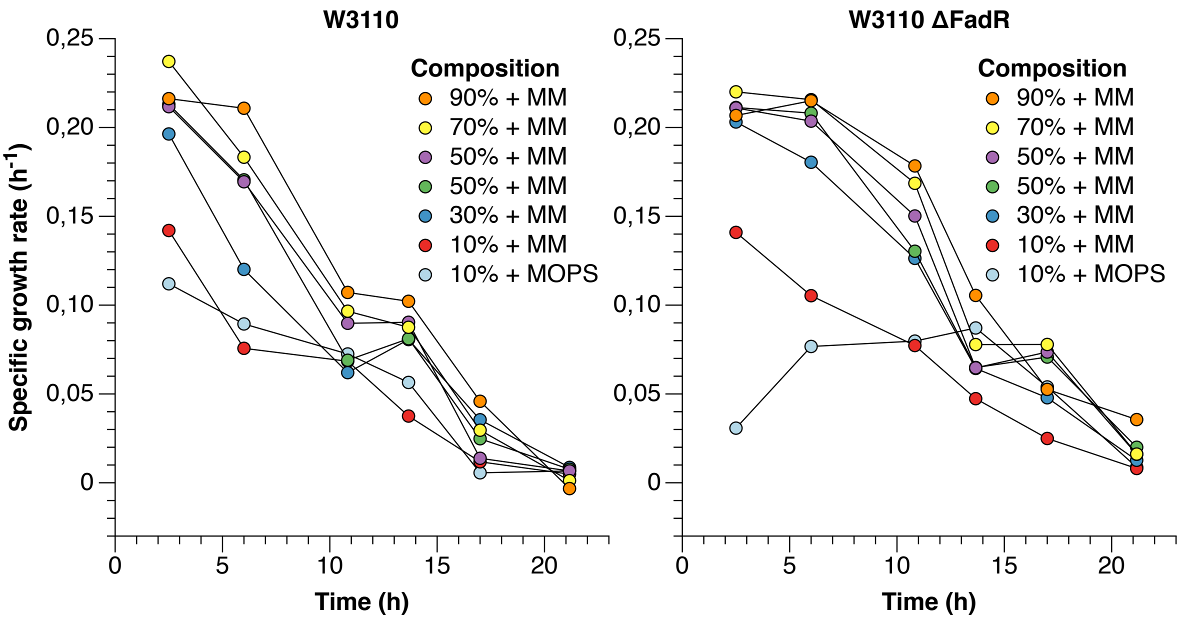

Fig. S1 Specific growth rates from the experiment presented in Fig. 1A, with the same colour coding

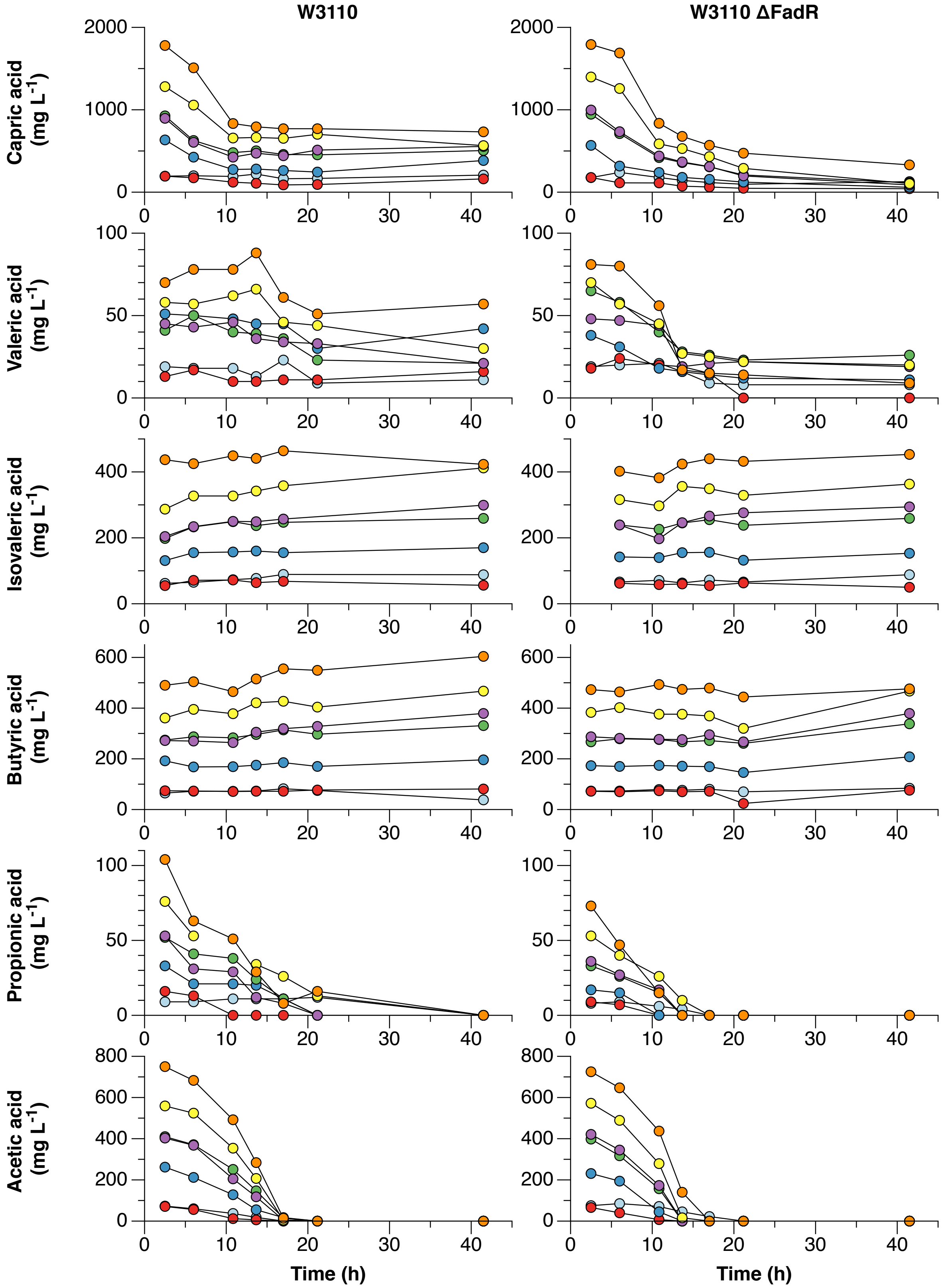

Fig. S2 Concentrations of all quantified volatile fatty acids from the experiments presented Fig. 1A, with the same colour coding
